# Glycyrol targets Pneumolysin (PLY) oligomerization to reduce *Streptococcus pneumoniae* toxicity

**DOI:** 10.3389/fphar.2024.1478135

**Published:** 2024-12-03

**Authors:** Yudi Li, Hongji Wu, Yibo Hu, Haoji Meng, Yan Xu

**Affiliations:** ^1^ Department of Pediatrics, The First Affiliated Hospital of Henan University of Chinese Medicine, Zhengzhou, Henan, China; ^2^ School of Pediatrics, Henan University of Chinese Medicine, Zhengzhou, Henan, China

**Keywords:** glycyrol, PLY, *Streptococcus pneumoniae*, TCM, toxicity

## Abstract

**Aim of the study:**

Exploring the potential of glycyrol to reduce the invasiveness of *Streptococcus pneumoniae* (*S. pneumoniae*).

**Materials and Methods:**

Cell experiments were performed using A549 alveolar epithelial cells and *S. pneumoniae* D39. Glycyrol was added to A549 cells mixed with or without Pneumolysin (PLY) to detect the effect of Glycyrol on PLY toxicity. Glycyrol was used to detect the effect on S. pneumoniae toxicity and PLY production. Mice was used to detect the anti-infectious ability of Glycyrol to regulate *S. pneumoniae* infection. Western blot and Molecular docking were used to detect how and where Glycyrol inhibits PLY toxicity.

**Results:**

We discovered that glycyrol, a main component of the widely recognized Chinese herbal medicine licorice, reduce the virulence of PLY in *S. pneumoniae* invasion; glycyrol achieves this effect by interacting with PLY through hydrogen bonding, van der Waals interactions, and solvation effects to reduce the pore-forming toxicity of PLY. Moreover, glycyrol did not affect the growth of *S. pneumoniae* or the production of PLY.

**Conclusion:**

We have actually discovered that Glycyrol, a major component of the widely known Chinese herbal medicine *Glycyrrhiza uralensis* Fisch., interacts with PLY through hydrogen bonds, Van der Waals and solvation to reduce the pore-forming toxicity of PLY and the toxicity of *S. pneumoniae* invasion, while not affecting the growth of *S. pneumoniae* and the production of PLY.

## 1 Glycyrol targets PLY to inhibit *Streptococcus pneumoniae*

In traditional Chinese medicine, it is believed that man and nature develop harmoniously and are co-dependent, and an imbalance on one side will greatly harm the other side. For example, the relationship between humans and bacteria seems to be imbalanced. Since the discovery of penicillin in the 1920s, many antibiotics have been used in industrialized and emerging areas, and some antibiotics are used as growth supplements and growth promoters for animals. Data obtained in China showed that veterinary antibiotics comprise up to 84.3% of antibiotics administered, and more than ten kinds of antibiotics have been detected in one type of livestock (Fang, 2016). Antibiotics that are widely used for veterinary drugs, livestock and poultry feed additives, and fruit and crop production also enter the soil‒plant system directly or indirectly, which is the most important environments for human and animal life. After antibiotics and their metabolites enter the soil environment, they can remain active in the soil for a long time. Similar to those of veterinary antibiotics, antibiotic-resistant bacteria (ARB)/resistance genes (ARGs) in the environment gradually migrate into plant systems. Ultimately, this process directly or indirectly increases the risk that human pathogenic bacteria become resistant to drugs through the food chain, threatening human public health and safety (Huang et al., 2020). In response, many studies have been conducted on nanotechnology (Rashki et al., 2021), phages (Hatfull et al., 2022), antimicrobial peptides (Luo and Song, 2021), probiotics (Zhang et al., 2022) and other methods. However, the mass production details, specificity, and specific mechanisms of these methods remain unclear. Therefore, much time will be needed for the application of the above research in large-scale clinical practice.

However, Chinese medicine is different. As a treatment method that has been used clinically for thousands of years, Chinese medicine has long been generally accepted by the Asian population. Uncovering the new antibacterial, bacteriostatic or resistance-reducing mechanisms of traditional Chinese medicines involves exploring the different effects of medicinal materials already on the market, and the products do not induce anxiety in the general population. The application of traditional Chinese medicine is guided by theory but certain rules must be followed. Usually, the effects that clear heat and detoxify the body are similar to the effects that kill bacteria. Among these drugs, *Glycyrrhiza uralensis* Fisch. (wfo-0000186028 in World Flora Online) is special, as it is a traditional Chinese medicine with various properties and is among the most commonly used drugs in clinical traditional Chinese medicine. According to the preparation method, raw Glycyrrhiza uralensis Fisch. can clear heat, detoxify the body, moisten the lungs, relieve cough symptoms, and reconcile various medicinal properties; roasted Glycyrrhiza uralensis Fisch. can replenish the spleen and replenish qi. Tao Hongjing, a medical scientist in the Southern Dynasties, revered Glycyrrhiza uralensis Fisch. as the “old man of the country”, which is a title of an emperor’s teacher. He believed that “this herb is the king of all medicines” and can regulate the toxicity and side effects of many drugs. To treat asthma and cough symptoms, Glycyrrhiza uralensis Fisch. can be used alone or in combination with other drugs. However, unlike most Chinese herbal medicines, the mechanism of Glycyrrhiza uralensis Fisch. has not been elucidated. It is important to identify effective components in complex traditional Chinese medicine for further research because the development of traditional Chinese medicine is restricted when these components are unknown. Due to the importance of Glycyrrhiza uralensis Fisch. in many herbal medicines and the evidence that Glycyrrhiza uralensis Fisch. exhibits antibacterial effects (He et al., 2006), we believe that the research significance and value of Glycyrrhiza uralensis Fisch. in traditional Chinese medicine are very high.

Among the complex components of *G. uralensis* Fisch., glycyrol is notable as it performs many active functions. Glycyrol has been found to induce cell cycle progression, apoptosis, and autophagy (Xu and Kim, 2014) and to regulate inflammatory responses induced by *Candida* spp. (Rhew and Han, 2016) and LPS(Xu and Kim, 2014). Glycyrol can even regulate immune responses by inhibiting calcineurin activity (Li et al., 2010). Additionally, based on our previous research on *Streptococcus pneumoniae*-related lung inflammation (Guo et al., 2021), we interested in determining if glycyrol is the active ingredient in Glycyrrhiza uralensis Fisch. that plays the main antibacterial role. Therefore, in this study, we investigated the antibacterial activity of glycyrol, exploring its ability to reduce the inflammatory response, and its ability to inhibit the oligomerization of pneumolysin through mouse and cell experiments.

## 2 Materials and methods

### 2.1 Cells, bacteria and animals

A549 alveolar epithelial cells were purchased from ATCC (Manassas, VA, United States) and grown in DMEM (Gibco Life Technologies, Inc., Grand Island, NY, United States) supplemented with 10% fetal bovine serum (FBS; Biological Industries, Israel). *S. pneumoniae* D39 serotype 2 (NCTC7466) (a gift from Professor Huang Jian of Zunyi Medical University, Guizhou, China) was cultured in THY media in an incubator at 37°C and 5% CO2. BALB/c mice (female, 6–8 weeks old, 20–22 g) were purchased from Liaoning Changsheng Biotechnology Co., Ltd. (Experimental Animal Production License Number: No. SCXK (Liao) 2020-0001) and kept at 24°C ± 3°C, a relative humidity of 40% ± 5%, a light/dark alternating cycle, noise <55 db, free access to water and food, and the replacement of litter twice a week. The animal experiments were approved by the Experimental Animal Welfare and Ethics Committee of Henan University of Traditional Chinese Medicine (IACUC-202404012).

### 2.2 Drugs

**FIGURE 1:**
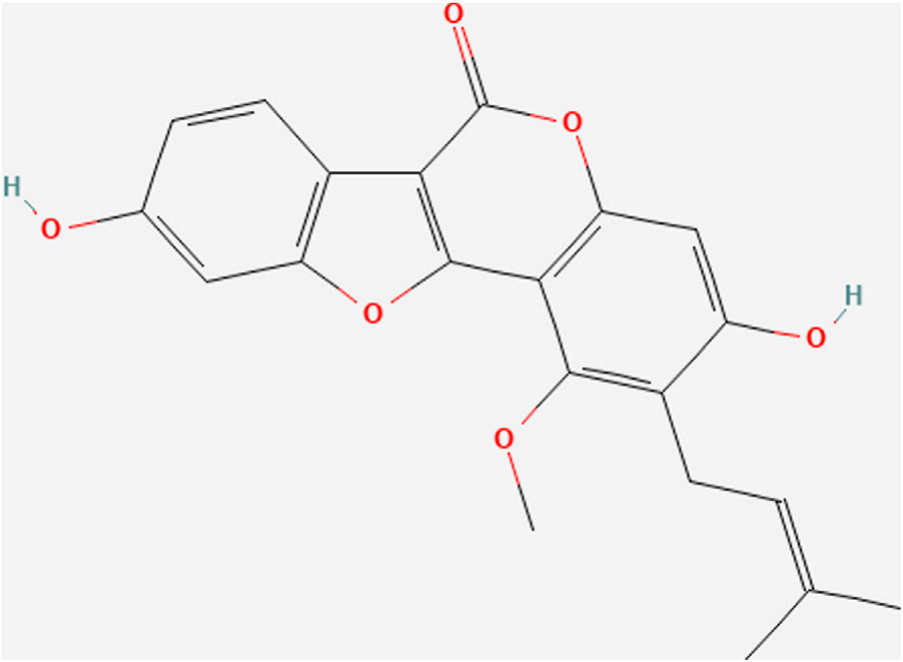
Glycyrol structure.

Glycyrol (Figure 1, CAS No: 23013-84-5, purity >98%) was purchased from Chengdu Phytochemical Pure Biotechnology Co., Ltd. After adding PBS, it was prepared into a stock solution of 128 μg/mL and stored at 4°C;

Todd-Hewitt Broth (THB), dimethyl sulfoxide (DMSO), skim milk powder, trypsin, imidazole, tetramethylethylenediamine (TEMED) and β-mmercaptoethanol (β-ME) were purchased from Sigma‒Aldrich; a BCA protein quantification kit and an enhanced chemiluminescence (ECL) solution were purchased from Thermo Fisher; and horseradish peroxidase (HRP)-labeled goat anti-mouse secondary antibody was purchased from Protech. Polyvinylidene fluoride (PVDF) membranes were purchased from Roche, a live/dead cell viability detection kit was purchased from Invitrogen, and fetal bovine serum albumin (fetal bovine serum; FBS) was purchased from Biological Industries. Thirty percent acrylamide, ammonium persulfate, sodium dodecyl sulfate (SDS), and disodium ethylenediaminetetraacetate (EDTA) were purchased from Dingguo Changsheng Biotechnology Co., Ltd.

### 2.3 PLY and hemolysis experiments

Pneumolysin (PLY) was purchased from Fitzgerald Company (80R-4390) in the United States. Different concentrations of glycyrol (0, 4, 8, 16, 32, and 64 μg/mL) were mixed with PLY and incubated in PBS at 37°C for 30 min. Then, 25 μL of defibrinated sheep red blood cells was added to the mixture and incubated at 37°C for 10 min. After the final incubation, the mixture was centrifuged at 3,000 *g* for 5 min, and the hemolytic activity of the supernatant was measured using a microplate reader at OD543 nm.

### 2.4 Antibacterial activity and antibacterial curve

Glycyrol antibacterial curves were generated via the microbroth method (Qiu et al., 2012), and different concentrations of glycyrol (0, 4, 8, 16, 32, and 64 μg/mL) and *S. pneumoniae* were cultured in THB. The growth of *S. pneumoniae* was monitored every 60 min with a UV spectrophotometer at 600 nm.

### 2.5 A549 cell cytotoxicity assay

In a 96-well plate, 2 × 10^4^ A549 cells were added to each well and incubated overnight. Then, 3.0 μL of glycyrol-pretreated PLY was added at different concentrations, and the samples were placed in a 37°C incubator for 5 h. Cells were processed using a live/dead (green/red) staining kit (Invitrogen, Carlsbad, CA, United States) according to the manufacturer’s instructions, and cell viability was observed using a confocal laser scanning microscope (Olympus, Tokyo, Japan).

### 2.6 Western blot analysis

*Streptococcus pneumoniae* D39 was cultured in THY at 37°C, and different concentrations of glycyrol were added. After centrifugation (3,000 rpm, 10 min), the 5× SDS‒PAGE supernatant was incubated at 100°C for 10 min, separated by 12% SDS‒PAGE, transferred to a PVDF membrane, and blocked with 5% skim milk powder at room temperature for 2 h.

A monoclonal antibody against pneumolysin (1:1,000; Abcam, Cambridge, United Kingdom) was incubated overnight at 4°C. After the membrane was washed with PBST, secondary antibody (1:2000; Proteintech, Chicago, IL, United States) was added, and the membrane was incubated at 37°C for 1 h. After the membrane was washed with PBST, the color was developed using a Tanon-4200 imager (Tanon, Shanghai, China) and ECL reagent (Thermo Scientific, Rockford, IL, United States).

The methods used to detect oligomers and monomers were the same as above. Different concentrations of glycyrol and PLY were incubated at 37°C for 1 h and then boiled at 50°C for 10 min (5× SDS‒PAGE loading buffer without β-mercaptoethanol was added). PLY oligomers and monomers were then detected.

### 2.7 Mouse model of *Streptococcus pneumoniae* infection

*Streptococcus pneumoniae* D39 was cultured in THY at 37°C to mid-log phase (OD600 nm = 0.4), washed three times by centrifugation with PBS, and resuspended in PBS. Mice were anesthetized with light ether, and 1.5 × 10^8^ colony-forming units (CFU) were inoculated into the left nostril of the mice for lung infection. Infected mice (n = 10) were injected subcutaneously with glycyrol (64 μg/mL, 20 mg/kg) or DMSO every 8 h.

Mice (n = 10) were sacrificed 48 h after infection. Bronchoalveolar lavage fluid from mice was collected and centrifuged using an ELISA kit (eBioscience, San Diego, CA, United States) to determine the levels of cytokines (IL-1β, IL-6, and TNF-α). The left lung of each mouse was removed, the overall changes in the lung were observed, and pictures were taken to collect images. Then, the sections were fixed with 10% formalin solution for 24 h, dehydrated with gradient concentrations of ethanol (70%, 80%, 90%, 95% and 100%), cleared with xylene, embedded in paraffin, sectioned, dewaxed with xylene and ethanol, subjected to HE staining, and mounted. Finally, histopathological changes were observed under a light microscope, and images were collected. Filter paper was used to absorb moisture from the surface of the other lung tissues, which were then weighed and placed in a 4 mL sterile centrifuge tube. An electric tissue grinder was used to fully grind the lung tissue, 900 mL of sterile PBS was added to resuspend the tissue cells, 100 μL of 1% Triton X-100 was added, and the mixture was shaken and mixed for 5 min to fully lyse the cells. After 10-fold gradient dilution of the sample, 10 μL was spread on a blood agar plate and cultured at 37°C and 5% CO for 24 h, after which the number of colonies was counted. The animal experiments were approved by Laboratory Animal Welfare and Ethics Committee of HenanUniversity of Traditional Chinese Medicine (IACUC-202404012).

The dry and wet weights of the lung tissue were measured, and the wet/dry weight ratio was calculated.

### 2.8 Molecular docking

A molecular docking study was performed to investigate the binding mode between glycyrol and *S. pneumoniae* pneumolysin using AutoDock vina 1.1.2. The three-dimensional (3D) structure of pneumolysin (PDB ID: 4QQA) was downloaded from the RCSB Protein Data Bank (www.rcsb.org). The 3D structure of glycyrol was drawn with ChemBioDraw Ultra 14.0 and ChemBio3D Ultra 14.0 software. The AutoDockTools 1.5.6 package was used to generate the docking input files. The binding sites of the pneumolysin were identified as center_x: 15.338, center_y: 65.624, and center_z: 30.303 with dimensions size_x: 15, size_y: 15, and size_z: 15, respectively. To increase the docking accuracy, the exhaustiveness value was set to 16. For Vina docking, the default parameters were used unless otherwise mentioned. Then, an MD study was performed to revise the docking results.

### 2.9 Molecular dynamics

The Amber 14 and AmberTools 15 programs were used to perform MD simulations of the selected docked pose. Aloin was first prepared by ACPYPE, a tool based on ANTECHAMBER for generating automatic topologies and parameters in different formats for different molecular mechanics programs, including the calculation of partial charges. Then, the forcefield “leaprc.gaff” (generalized amber forcefield) was used to prepare the ligand, while “leaprc.ff14SB” was used for the receptor. The system was placed in a rectangular box (with a 10.0 Å boundary) of TIP3P water using the “SolvateOct” command with the minimum distance between any solute atoms. Equilibration of the solvated complex was performed by performing a short minimization (2000 steps of each steepest descent and conjugate gradient method), 1,000 ps of heating, and 500 ps of density equilibration with weak restraints using the GPU (NVIDIA^®^ Tesla K20c) accelerated PMEMD (Particle Mesh Ewald Molecular Dynamics) module. Finally, 40 ns of MD simulations were carried out.

### 2.10 Binding free energy and energy decomposition per residue calculations

The binding free energies (ΔG_bind_ in kcal/mol) were calculated using the Molecular Mechanics/Generalized Born Surface Area (MM/GBSA) method, implemented in AmberTools 15. Moreover, to identify the key protein residues responsible for the ligand binding process, the binding free energy was decomposed on a per-residue basis. For each complex, the binding free energy of MM/GBSA was estimated as follows:

$$\Delta G_{\text{bind}}=G_{\text{complex}}‒G_{\text{protein}}‒G_{\text{ligand}}$$

where ΔG_bind_ is the binding free energy and G_complex_, G_protein_ and G_ligand_ are the free energies of the complex, protein, and ligand, respectively.

### 2.11 Statistical analysis

The data are expressed as the mean ± standard deviation (S.D.) (n = 10) and were analyzed via GraphPad Prism 6.0 (GraphPad Software) using two-tailed independent-sample t tests and one-way ANOVA followed by Tukey’s *post hoc* multiple comparison test. *, *p* < 0.05.

## 3 Results

### 3.1 Glycyrol can directly reduce the inflammatory response in the lungs of mice infected with *Streptococcus pneumoniae*

Pneumonia caused by *S. pneumoniae* infection shows extremely high severity and pathogenicity in animal models. In order to comprehensively evaluate the severity of lung inflammation and determine the invasion of pathogens, three core detection indicators are usually relied on: visual observation of lung pathological changes, HE staining pathological analysis of lung tissue, and accurate determination of the bacterial content of lung tissue. Given the importance of these indicators, this study first and systematically carried out these three aspects of detection.

**FIGURE 2:**
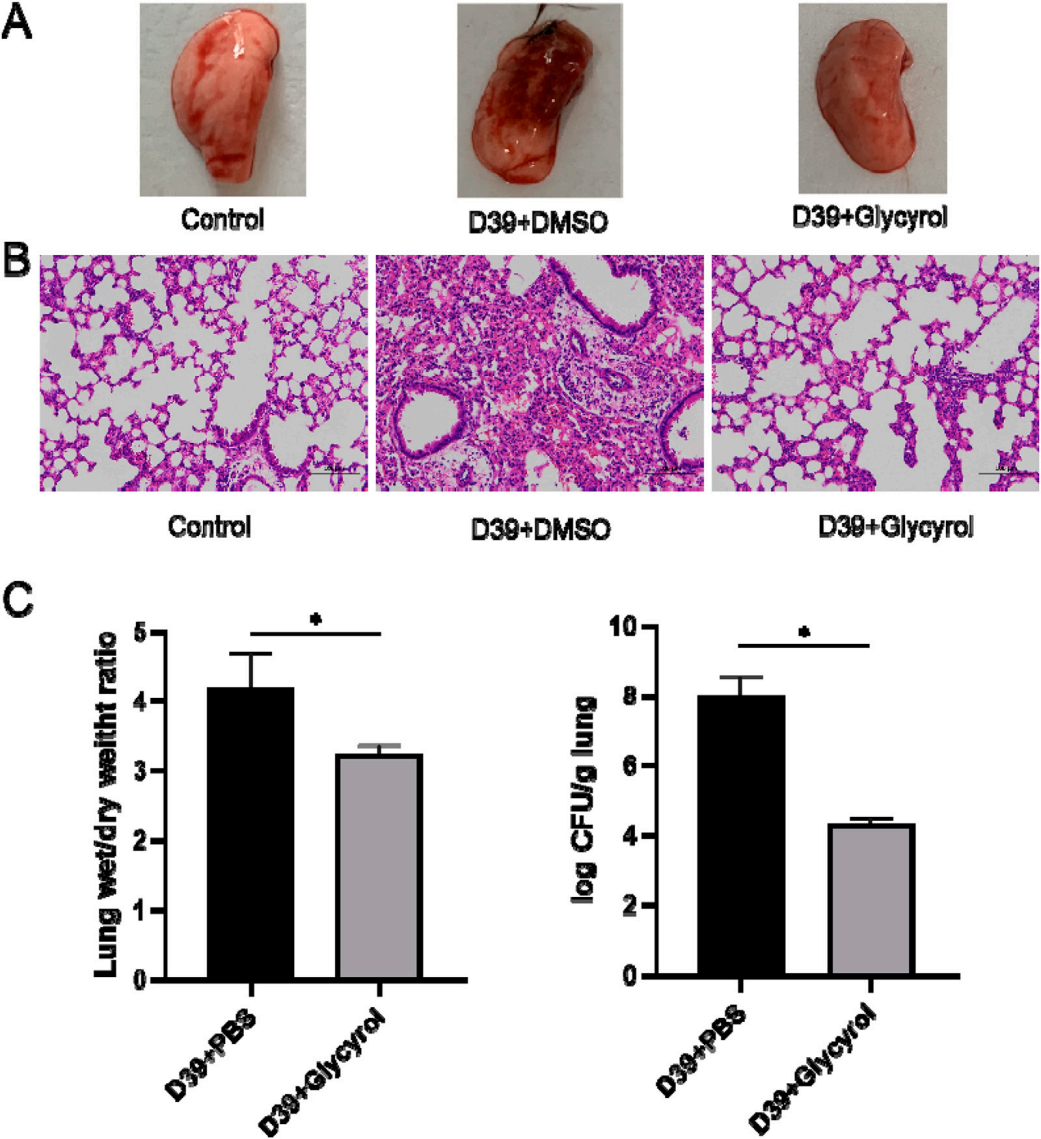
Glycyrol can directly reduce the inflammatory response and bacterial content in the lungs of mice infected with *Streptococcus pneumoniae*. **(A)** Macroscopic observation of the lungs of mice treated with Glycyrol. **(B)** Histopathological examination of the lungs of mice treated with Glycyrol. **(C)** Detection of the wet/dry ratio of the lungs and the bacterial content in the lungs. *, *P* < 0.05.

First, we used intranasal instillation of *S. pneumoniae* D39 to establish a pneumonia infection model. As shown in Figure 2A, D39 aggravated lung infection, while glycyrol completely reversed the inflammatory response. The same result was also obtained through the pathological examination shown in Figure 2B. Moreover, the degree of pulmonary edema in mice infected with D39 also increased (Figure 2C), and glycyrol reduced the degree of pulmonary edema. Additionally, we tested the customized number of *S. pneumoniae* in mouse lungs. After weighing and grinding the samples, performing cell lysis and diluting the mouse lungs with a gradient dilution, we spread the samples on blood agar plates and counted the number of colonies. The results are shown in Figure 2C. The number of bacteria that colonized the lungs of mice in the positive control group infected with D39 reached 8 CFUs/g after the log value was obtained. After glycyrol treatment, the number of bacteria that colonized the mice lungs decreased to 4 CFUs/g. This result shows that glycyrol can significantly reduce the inflammatory response and bacterial colonization number in the lungs of mice infected with *S. pneumoniae*.

### 3.2 Glycyrol can directly reduce the expression of inflammatory factors in the alveolar lavage fluid of mice

**FIGURE 3:**
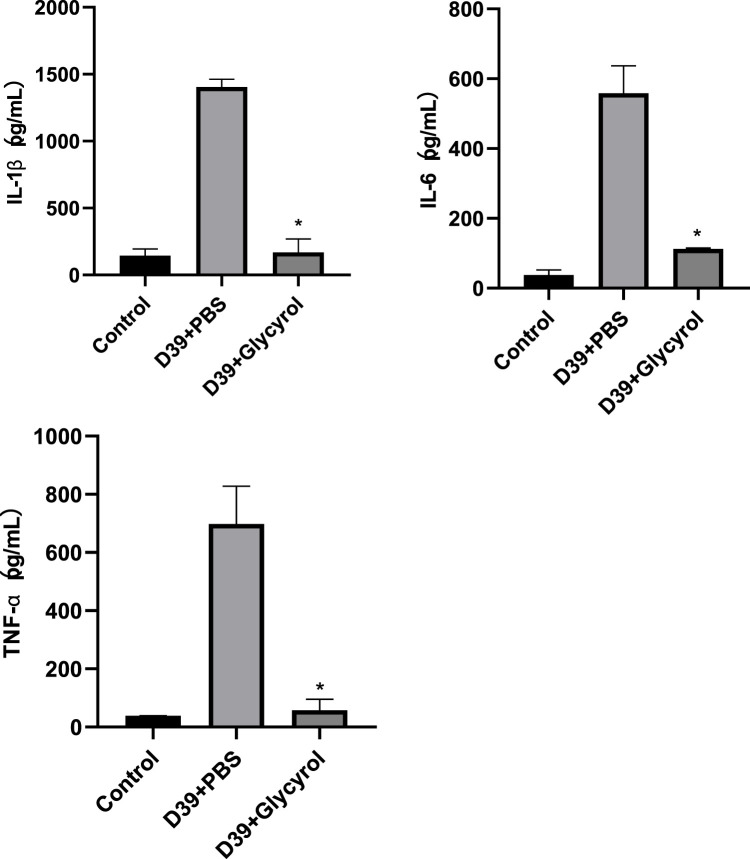
Glycyrol reduces the expression of inflammatory IL-1β, IL-6, and TNF-α.* Compared with D39+PBS group, *P* < 0.05.

Cytokine storm syndrome is a phenomenon in which the level of proinflammatory cytokines increases sharply after the body is stimulated by microorganisms or drugs, causing immune system disorders (Jarczak and Nierhaus, 2022). The resulting disruption in the proinflammatory and anti-inflammatory balance and the intense self-reinforcement of various feedback mechanisms ultimately lead to systemic damage, multiorgan failure, or death. Bacterial invasion is a common mechanism that leads to increases in the levels of proinflammatory factors. The resulting disruption of the proinflammatory and anti-inflammatory balance and the intense self-reinforcement of various feedback mechanisms ultimately lead to systemic damage, multiorgan failure, or death. Bacterial invasion is a common mechanism that leads to an increase in proinflammatory factors. If glycyrol can reduce local inflammation, it should also reduce the release of proinflammatory factors, which was observed in the experiment. Alveolar lavage fluid was collected for IL-1β, IL-6, and TNF-α testing, and it was found that 64 μg/mL of Glycyrol could significantly reduce the release of these proinflammatory factors, and there was a significant difference compared with that after D39 infection, as shown in Figure 3.

### 3.3 Glycyrol reduces PLY-mediated erythrocyte rupture and A549 cell death

**FIGURE 4:**
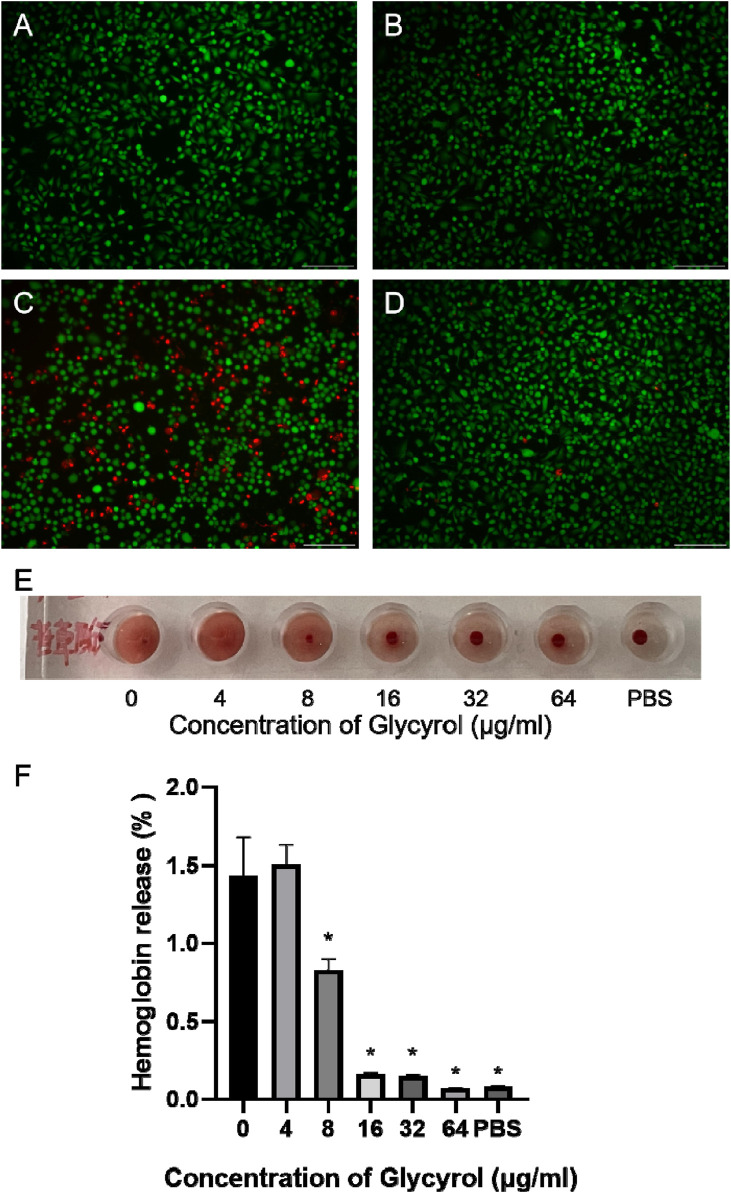
Glycyrol reduces PLY-mediated erythrocyte rupture and A549 cell death. **(A)** is untreated, **(B)** is 64 glycyrol, **(C)** is PLY, **(D)** is 64 glycyol and PLY coincubated, and **(E)** and **(F)** are the hemolysis conditions observed after PLY and glycyrol were mixed. *Compared with the 0 μg/mL group, *P* < 0.05.

Since PLY is the main virulence factor of *S. pneumoniae*, it participates in bacterial transmission, colonization, and invasion (Weiser et al., 2018). As PLY performs multiple biological activities, such as cell lysis and DNA damage, targeting PLY has been considered an alternative strategy for the treatment of *S. pneumoniae* infection (Qi et al., 2021). PLY exhibits cell-destroying properties, and we used PLY as a research object to detect Glycyrol-mediated hemolysis of red blood cells through hemolysis experiments. When glycyrol was not added, PLY completely mediated the hemolysis of red blood cells, while 16 μM, 32 μM, and 64 μM completely reversed the hemolysis process (Figures 4E, F). A549 cells were further used for live-dead cell staining, and 64 μM glycyrol also reduced the PLY-mediated death of A549 cells (Figures 4A–D). The above results demonstrated at the cellular level that Glycyrol can reduce PLY-mediated A549 cell death and erythrocyte hemolysis, which means that the target of Glycyrol seems to be PLY, the main virulence factor of *S. pneumoniae*, rather than the entire *Streptococcus* pneumoniae strain.

### 3.4 Glycyrol does not inhibit the growth of *Streptococcus pneumoniae* but can reduce the rate of PEL

**FIGURE 5:**
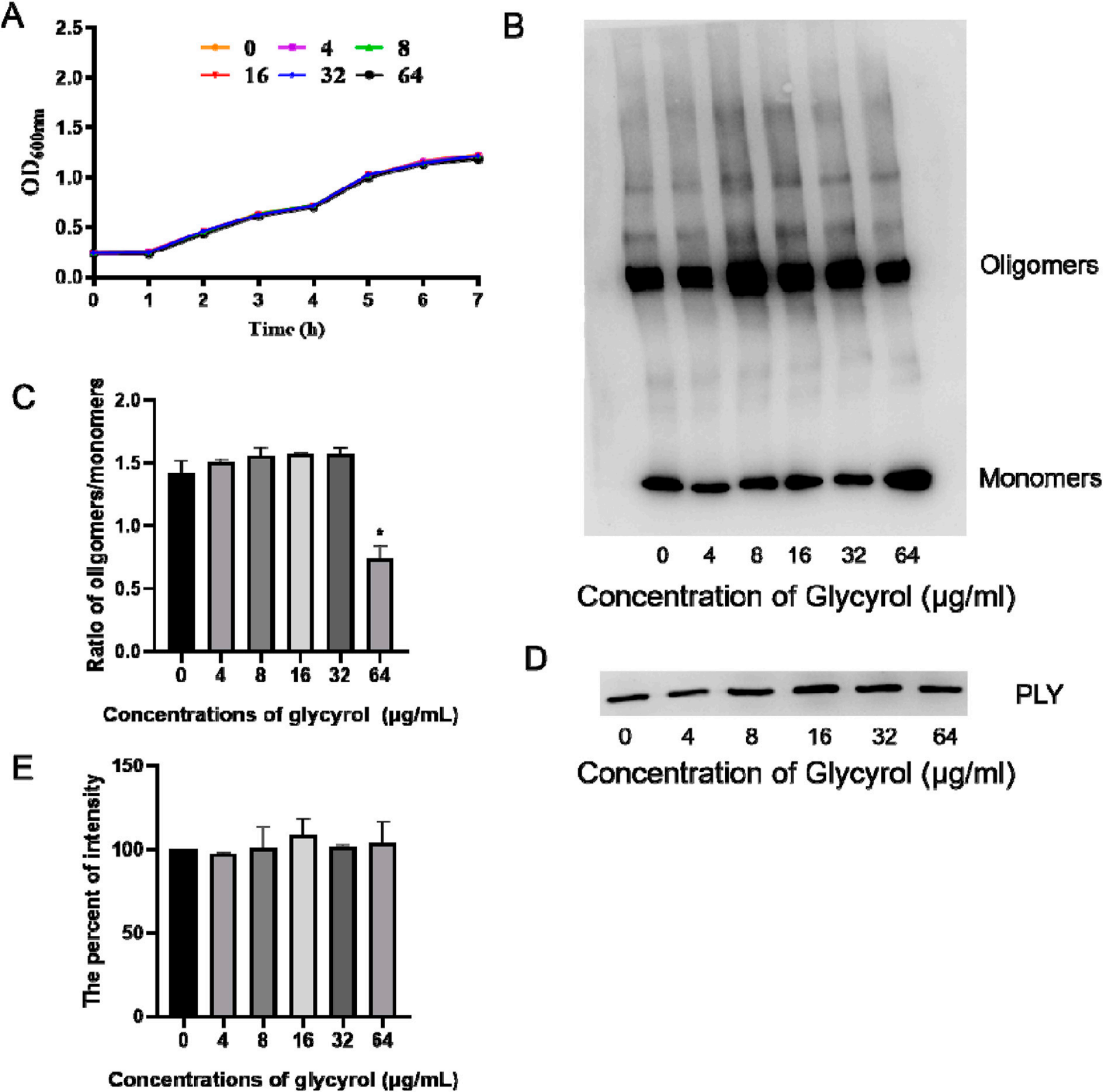
Effect of glycyrol on the bacterial growth curve, PLY production and oligomerization. **(A)** The influence of glycyrol on the growth of *Streptococcus pneumoniae*. *Streptococcus pneumoniae* was cultured to an OD600 nm of 0.25 in THB and then cocultured with the indicated concentrations of glycyrol at 37°C. Then, the OD600 nm of each sample was monitored every 30 min. **(B)** PLY was pretreated with various concentrations of glycyrol,and then, the PLY monomers or oligomers were assessed by immunoblot analysis. **(C)** Intensity for the ratio of oligomers/monomers measured using ImageJ *, compared with the group without glycyrol, P< 0.05. **(D)** *S. pneumoniae* was cocultured with the indicated concentrations of glycyrol, and the PLY in the supematants was detected by immunoblot analysis. **(E)** The percent intensity of each sample was measured using ImageJ software by comparison with the sample without glycyrol. Data are presented as the mean ± SD (n ≥ 3).

To verify whether the target of Glycyrol is PLY or the entire *S. pneumoniae* strain, we tested the MIC of Glycyrol and found that Glycyrol had no effect on the growth of *S. pneumoniae* (Figure 5A). In other words, Glycyrol does play a therapeutic role by targeting PLY, the main virulence factor of *S. pneumoniae*.

PLY is actually composed of four domains that form a complex spatial structure. It can form helical oligomers at room temperature. This oligomer presents a curved protein conformation similar to the pore state. The pore-forming activity of PLY is also the main factor in the virulence of *Streptococcus pneumoniae*. Based on the above facts, we speculate that the mechanism of action of Glycyrol is to directly reduce the process of PLY forming a pore structure.

To verify the hypothesis, we first found that Glycyrol could not reduce the production of PLY (Figures 5D, E). We continued to detect the effect of Glycyrol on PLY oligomerization by Western blot. As shown in Figures 5B, C, we found that Glycyrol could reduce the process of PLY forming a pore structure. Therefore, the hypothesis was verified that Glycyrol reduced the toxicity of *S. pneumoniae* by reducing the pore formation process of PLY, and at the same time did not affect the bacterial activity or PLY production.

### 3.5 Molecular dynamics results

**FIGURE 6:**
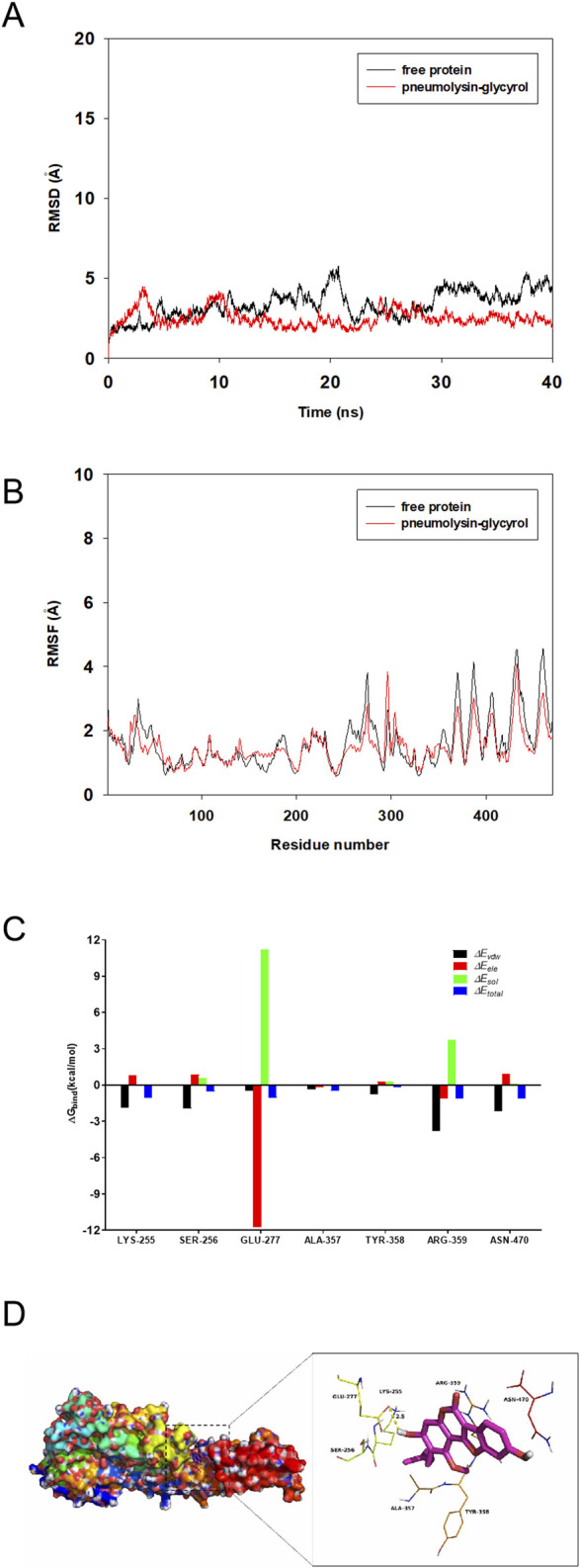
Glycyrol and the PLY, molecular docking and molecular dynamics simulations **(A)**. The root-mean-square deviations (RMSDs) of all atoms in the PLY-glycyrol complex with respect to its initial structure as a function of time. **(B)**. RMSF of residues of the whole protein in the PLY-glycyrol complex and free PLY during the 40 ns simulation. **(C)**. Decomposition of the binding energy on a per-residue basis in the PLY-glycyrol complex. **(D)**. The predicted binding mode of glycyrol in the PLY binding pocket obtained from MD simulations.

To explore the potential binding mode between glycyrol and PLY, molecular docking and molecular dynamics simulations were performed using the AutoDock vina 1.1.2 and Amber14 software packages. The binding mechanism of PLY with glycyrol was determined by 40-ns molecular dynamics simulations based on the docking results. Next, the root-mean-square deviation (RMSD) values of the protein backbone were calculated based on the starting structure during the simulation time and are plotted in Figure 6A; this was performed to explore the dynamic stability of the complex and ensure the rationality of the sampling strategy. The protein structures of the two systems were stabilized during the 40-ns simulation.

The root mean square fluctuations (RMSFs) were calculated for the residues of the whole protein in the PLY-glycyrol complex and in the free PLY to reveal the flexibility of the residues. The RMSFs of these residues are shown in Figure 6B, clearly depicting different flexibilities in the binding site of PLY in the presence and absence of glycyrol. All of the residues in the PLY binding site that bind with glycyrol showed a small degree of flexibility, with an RMSF of less than 3 Å compared with that of free PLY; therefore, these residues seem to be more rigid as a result of binding to glycyrol. However, the residues at the C-terminus of PLY showed a large degree of flexibility, with RMSF values nearly reaching 5 Å compared with those of free glycyrol; therefore, that these residues seem to be more flexible as a result of binding to glycyrol.

To gain more information on the residues surrounding the binding site and their contributions to the system, the electrostatic, *van der Waals*, solvation, and total contributions of the residues to the binding free energy were calculated with the MMGBSA method. The summations of the per residue interaction free energies were separated into *van der Waals* (∆*E*_*vdw*_), solvation (∆*E*_*sol*_), electrostatic (∆*E*_*ele*_) and total contribution (∆*E*_*total*_) values. In the PLY-glycyrol complex, Glu-277 has an excellent electrostatic (∆*E*_*ele*_) contribution, with a value of < −11.0 kcal/mol (Figure 6C). Detailed analysis revealed that residue Glu-277 is close to the hydroxyl group of glycyrol, leading to a hydrogen bond between PLY and glycyrol (Figure 6D). Moreover, residue Asn-470, with a ∆*E*_*vdw*_ of < −2.0 kcal/mol, formed appreciable *van der Waals* interactions with glycyrol due to the close proximity between the residues and glycyrol. Except for the interaction of residue Asn-470, most decomposed energy interactions originated from *van der Waals* interactions and involved hydrophobic interactions (*i.e.,* Ala-357). In addition, the total binding free energy of the PLY-glycyrol complex was calculated according to the MMGBSA approach, and an estimated ∆G_*bind*_ of −19.6 kcal/mol was found for glycyrol, suggesting that glycyrol can strongly bind PLY and interact with the binding site of PLY.

In summary, a rational explanation of the interactions between glycyrol and PLY was obtained through molecular simulations, providing valuable information for the further development of PLY inhibitors.

## 4 Discussion

### 4.1 PLY is the main virulence protein of S. pneumoniae. Inhibition of PLY can reduce the toxicity of *Streptococcus* pneumoniae

Transmission, colonization, and invasion are the pathogenic processes of *Streptococcus* pneumoniae. PLY, as an important virulence factor, is involved in the entire pathogenic process (Weiser et al., 2018). First, the expression of PLY can increase the survival rate of *S. pneumoniae in vitro* (Zafar et al., 2017). After *S. pneumoniae* invades the human body, the release of PLY can reduce the ciliary beating of respiratory epithelial cells, thereby reducing the number of *S. pneumoniae* cleared by cilia, thereby facilitating the endocytosis of *S. pneumoniae* invading epithelial cells. At the same time, PLY also directly destroys epithelial cells. This rough method directly destroys the epithelial barrier. When *Streptococcus* pneumoniae breaks through the endothelium and enters the blood, PLY can also help *Streptococcus* pneumoniae effectively avoid phagocytosis by macrophages (Weiser et al., 2018).

PLY consists of four domains that form a complex spatial structure. A large number of PLY monomers can form helical oligomers at room temperature, and these oligomers exhibit a bent protein conformation similar to the pore state (Tilley et al., 2005). It can be inserted into the cell membrane to change the osmotic pressure inside and outside the cell, thereby causing the cell to rupture. In this study, Glycyrol directly acts on the process of PLY oligomerization and reduces the formation of pore structures, which can achieve the following effects: 1) Reduce the inhibitory effect of PLY on the cilia swing of epithelial cells and increase the clearance rate of respiratory *S. pneumoniae*; 2) Prevent PLY from destroying the epithelial barrier and reduce the entry of *S. pneumoniae* into the blood; 3) Inhibit the ability of *S. pneumoniae* to evade phagocytes, thereby helping the immune system to clear *S. pneumoniae*.

### 4.2 Glycyrol does not reduce the production of PLY and has no direct antibacterial effect

Due to the global increase in antimicrobial resistance, traditional treatments for bacterial infections have become ineffective (Vila et al., 2020). Classic methods for developing new antibacterial agents cannot sufficiently meet the current treatment needs. Neither changing the chemical structure of existing antibacterial agents to circumvent resistance mechanisms nor developing compounds that inhibit antibacterial resistance mechanisms has generated compounds that reached the clinical experimental stage (Alam et al., 2016).

Finding novel *in vitro* inhibitors of bacterial targets is extremely challenging due to difficulties in penetrating the bacterial cell wall. Although searching for new antibacterial compounds from human microbial metabolites through metagenomic and bioinformatics methods is theoretically feasible, this approach has been unsuccessful thus far (Theuretzbacher et al., 2019).

In addition, some nanoscale particles or materials may interact with protein and nucleic acid functional groups through oxidative stress induction, nonoxidative mechanisms, and the release of small amounts of metal ions. However, the cytotoxicity of nanoparticles must be overcome first (Seil and Webster, 2012).

Antimicrobial peptides help the host fight pathogens, and researchers generally believe that the mechanism of action involves membrane permeability; however, other mechanisms also occur, including inhibition of protein, DNA, and RNA synthesis and degradation of genetic material.

The activity of antimicrobial peptides is based on their composition and secondary structure, and over the past few decades, antimicrobial peptides have often failed in preclinical studies due to their low stability or high *in vivo* toxicity (Ageitos et al., 2017; Molchanova et al., 2017).

Although phage therapy is viewed as a potentially promising alternative to combat drug-resistant pathogens, there are still several obstacles that must be overcome (Domingo-Calap and Delgado-Martínez, 2018) One obstacle is pharmacokinetics because a high dose of bacteria is needed to eliminate a bacterial population (even a small population); thus, bacteria must replicate within the host cell to exert their bactericidal effect. In terms of the host response, the immune response generated by neutralizing antibodies (which are produced by bacteria) must also be considered, and the weak stability of enzyme preparations is also a major limitation (Díez-Martínez et al., 2015; Rohde et al., 2018).

The above findings show many methods to fight against bacteria are still incomplete. In our study, we used Glycyrol to explore a virulence blocker that does not inhibit bacterial growth and reduces PLY ligation without reducing the production of the main virulence factor PLY. In contrast to inhibiting bacterial pili (Pinkner et al., 2006), lipid A (Ouyang et al., 2018) limits adhesion, affects the extracellular matrix, and interferes with mature biofilms (Roy et al., 2018). Glycyrol directly acts on the secreted protein PLY of *S. pneumoniae* and has no effect on *S. pneumoniae*; however, this treatment method hardly causes drug resistance. As the bacteria are unaware that their virulence has been suppressed, this method does not rely on or affect the host’s immunity.

### 4.3 Glycyrol can interact with PLY through van der Waals (ΔEvdw), solvation (ΔEsol), electrostatic (ΔEele) and total contributions (ΔEtotal) forces

**FIGURE 7:**
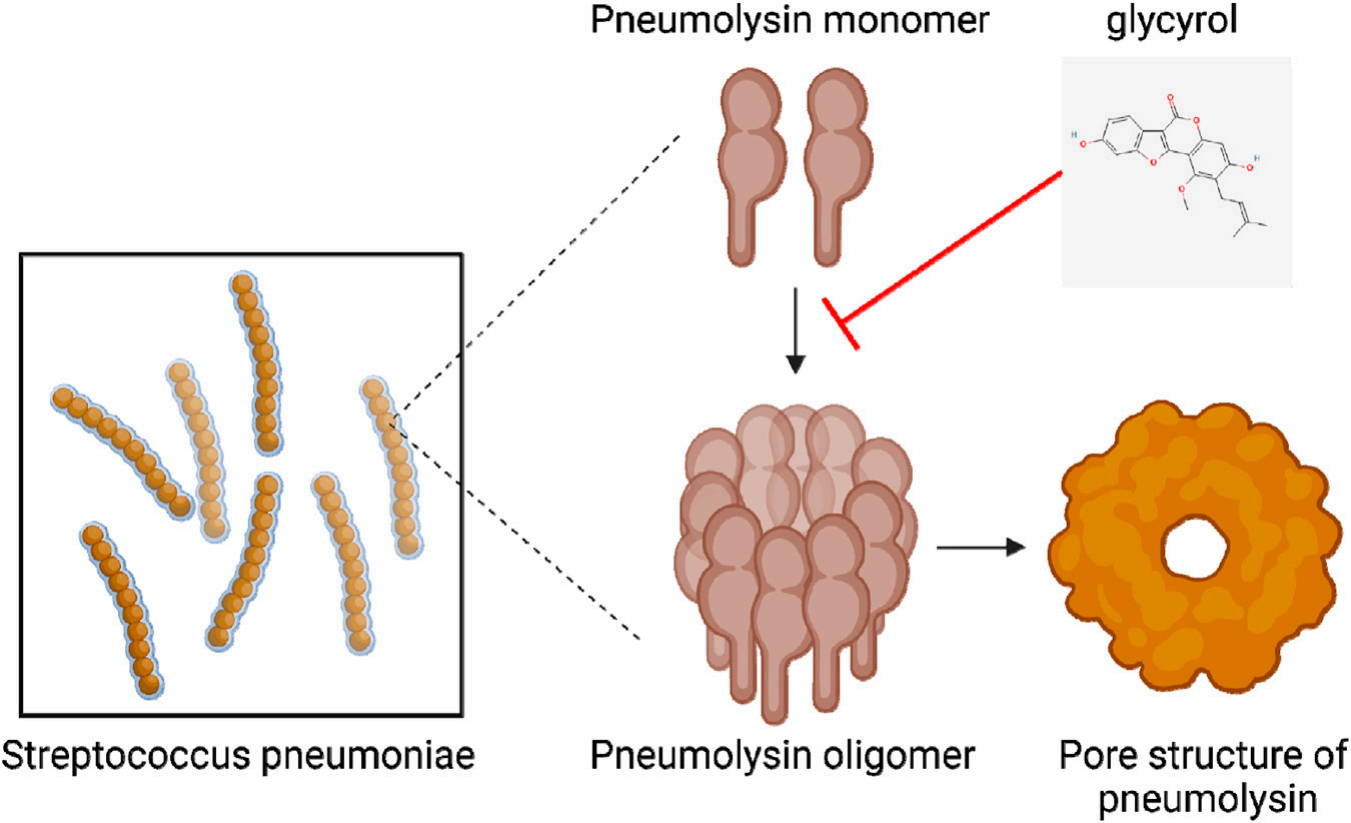
Glycyrol reduces the oligomerization of PLY into a pore-like structure through hydrogen bonding, Van der Waals and solvation interactions with PLY.

As a small molecule, PLY is a multifunctional protein composed of 471 amino acids with a molecular weight of 53 kDa. It is a member of the cholesterol-dependent cytolysin (CDC) family. PLY first forms an oligomerized prepore complex composed of 34–50 monomer molecules; then, the prepore complex undergoes a conformational change to form a β-barrel transmembrane pore with a diameter of approximately 25 nm. Glycyrol must interact with PLY to affect its function. Through a virtual screening method involving molecular docking technology, we found that glycyrol and PLY form hydrogen bonds and van der Waals and solvation interactions, especially at the Glu-277 site of PLY. Through the connection of these sites, we directly determined the molecular mechanism underlying the interactions between glycyrol and PLY. As shown in Figure 7. This interaction directly connects PLY with glycyrol via a molecular mechanism. Thus, we may be the first to explain the molecular mechanism by which glycyrol inhibits PLY oligomerization.

### 4.4 Traditional Chinese medicine plays an indispensable role in modern research, and glycyrol may become a new drug for the treatment of *Streptococcus pneumoniae* infection

As a complex mixture, traditional Chinese medicine has played a role in most modern research. Thus far, traditional Chinese medicine has been shown to affect ferroptosis, copper death, multiple inflammatory pathways, immune checkpoint regulation, and intestinal microbial metabolite regulation. It seems that every time a new regulatory mechanism of life is discovered, there are always Chinese medicines that can directly or indirectly regulate this new mechanism. Some scholars believe that the multicomponent and multitarget regulatory effects of traditional Chinese medicine lead to the continuous discovery of new mechanisms in traditional Chinese medicine. However, as a simple philosophical concept, traditional Chinese medicine and its underlying logic may describe the mechanisms that connect all aspects of life. In traditional Chinese medicine, there is always a balance. For example, if there is cold, there is heat; if there is an upper side, there is a lower side; if there is a liquid, there is a solid; and if there are bacteria, there will be ways to fight them. All things are interconnected and antagonistic. In traditional Chinese medicine, it is believed that this balance resulted from the evolution of life over billions of years. Therefore, it is understandable that in traditional Chinese medicine, diseases treatment involves the promotion or restraint of substances through the internal connection of animals, plants or their components.

In conclusion, we discovered that glycyrol, a main component of the widely recognized Chinese herbal medicine licorice, reduce the virulence of PLY in *S. pneumoniae* invasion; glycyrol achieves this effect by interacting with PLY through hydrogen bonding, van der Waals interactions, and solvation effects to reduce the pore-forming toxicity of PLY. Moreover, glycyrol did not affect the growth of *S. pneumoniae* or the production of PLY. We believe that glycyrol is a potential drug candidate that reduces the degree of inflammation after *S. pneumoniae* infection and reduces the toxicity of *S. pneumoniae* without leading to drug resistance.

## Data Availability

The original contributions presented in the study are included in the article/supplementary material, further inquiries can be directed to the corresponding author/s.

## Abbreviations

PLY, Pneumolysin; CFU, Colony-forming units; THB, Todd-Hewitt Broth; DMSO, dimethyl sulfoxide; TEMED, Tetramethylethylenediamine; β-ME, β-mmercaptoethanol; ECL, enhanced chemiluminescence; HRP, horseradish peroxidase; PVDF, Polyvinylidene fluoride; FBS, fetal bovine serum; SDS, sodium dodecyl sulfate; EDTA, ethylenediaminetetraacetate; RMSD, root-mean-square deviation; RMSFs, The root mean square fluctuations.

## References

[B1] Ageitos J. M. Sánchez-Pérez A. Calo-Mata P. Villa T. G. (2017). Antimicrobial peptides (AMPs): ancient compounds that represent novel weapons in the fight against bacteria. Biochem. Pharmacol. 133, 117–138. 10.1016/j.bcp.2016.09.018 27663838

[B2] Alam M. K. Alhhazmi A. DeCoteau J. F. Luo Y. Geyer C. R. (2016). RecA inhibitors potentiate antibiotic activity and block evolution of antibiotic resistance. Cell Chem. Biol. 23 (3), 381–391. 10.1016/j.chembiol.2016.02.010 26991103

[B3] Díez-Martínez R. De Paz H. D. García-Fernández E. Bustamante N. Euler C. W. Fischetti V. A. (2015). A novel chimeric phage lysin with high *in vitro* and *in vivo* bactericidal activity against Streptococcus pneumoniae. J. Antimicrob. Chemother. 70 (6), 1763–1773. 10.1093/jac/dkv038 25733585

[B4] Domingo-Calap P. Delgado-Martínez J. (2018). Bacteriophages: protagonists of a post-antibiotic era. Antibiot. (Basel). 7 (3), 66. 10.3390/antibiotics7030066 PMC6163168 30060506

[B5] Fang X. (2016). It’s time for the Chinese to say no to the abuse of antibiotics in animals and plants Chinese famous plant ecologist Jiang Gaoming talks about antibiotics in the ecological chain. Cap. Food Med. (1), 2.

[B6] Guo T. Guo Y. Liu Q. Xu Y. Wei L. Wang Z. (2021). The TCM prescription Ma-xing-shi-gan-tang inhibits Streptococcus pneumoniae pathogenesis by targeting pneumolysin. J. Ethnopharmacol. 275, 114133. 10.1016/j.jep.2021.114133 33892068

[B7] Hatfull G. F. Dedrick R. M. Schooley R. T. (2022). Phage therapy for antibiotic-resistant bacterial infections. Annu. Rev. Med. 73, 197–211. 10.1146/annurev-med-080219-122208 34428079

[B8] He J. Chen L. Heber D. Shi W. Lu Q. Y. (2006). Antibacterial compounds from Glycyrrhiza uralensis. J. Nat. Prod. 69 (1), 121–124. 10.1021/np058069d 16441081

[B9] Huang D. Y. M. Zhu Guofan S. M. Zhang Zhongyun Z. S. Bian Yongrong Q. J. Hu Feng J. X. (2020). Research progress on the migration, transformation and resistance reduction of antibiotics/resistant bacteria/resistant genes in the soil-plant system. Soil 52 (5), 10. 10.13758/j.cnki.tr.2020.05.004

[B10] Jarczak D. Nierhaus A. (2022). Cytokine storm-definition, causes, and implications. Int. J. Mol. Sci. 23 (19), 11740. 10.3390/ijms231911740 36233040 PMC9570384

[B11] Li J. Tu Y. Tong L. Zhang W. Zheng J. Wei Q. (2010). Immunosuppressive activity on the murine immune responses of glycyrol from Glycyrrhiza uralensis via inhibition of calcineurin activity. Pharm. Biol. 48 (10), 1177–1184. 10.3109/13880200903573169 20860439

[B13] Luo Y. Song Y. (2021). Mechanism of antimicrobial peptides: antimicrobial, anti-inflammatory and antibiofilm activities. Int. J. Mol. Sci. 22 (21), 11401. 10.3390/ijms222111401 34768832 PMC8584040

[B14] Molchanova N. Hansen P. R. Franzyk H. (2017). Advances in development of antimicrobial peptidomimetics as potential drugs. Molecules 22 (9), 1430. 10.3390/molecules22091430 28850098 PMC6151827

[B15] Ouyang P. He X. Yuan Z. W. Yin Z. Q. Fu H. Lin J. (2018). Erianin against *Staphylococcus aureus* infection via inhibiting sortase A. Toxins (Basel) 10 (10), 385. 10.3390/toxins10100385 30249042 PMC6215257

[B16] Pinkner J. S. Remaut H. Buelens F. Miller E. Aberg V. Pemberton N. (2006). Rationally designed small compounds inhibit pilus biogenesis in uropathogenic bacteria. Proc. Natl. Acad. Sci. U. S. A. 103 (47), 17897–17902. 10.1073/pnas.0606795103 17098869 PMC1693844

[B17] Qi Z. Guo Y. Zhang H. Yu Q. Zhang P. (2021). Betulin attenuates pneumolysin-induced cell injury and DNA damage. J. Appl. Microbiol. 130 (3), 843–851. 10.1111/jam.14769 32621771

[B18] Qiu J. Niu X. Dong J. Wang D. Wang J. Li H. (2012). Baicalin protects mice from *Staphylococcus aureus* pneumonia via inhibition of the cytolytic activity of α-hemolysin. J. Infect. Dis. 206 (2), 292–301. 10.1093/infdis/jis336 22551812

[B19] Rashki S. Asgarpour K. Tarrahimofrad H. Hashemipour M. Ebrahimi M. S. Fathizadeh H. (2021). Chitosan-based nanoparticles against bacterial infections. Carbohydr. Polym. 251, 117108. 10.1016/j.carbpol.2020.117108 33142645

[B20] Rhew Z. I. Han Y. (2016). Synergic effect of combination of glycyrol and fluconazole against experimental cutaneous candidiasis due to Candida albicans. Arch. Pharm. Res. 39 (10), 1482–1489. 10.1007/s12272-016-0824-7 27572154

[B21] Rohde C. Wittmann J. Kutter E. (2018). Bacteriophages: a therapy concept against multi-drug-resistant bacteria. Surg. Infect. (Larchmt) 19 (8), 737–744. 10.1089/sur.2018.184 30256176 PMC6302670

[B22] Roy R. Tiwari M. Donelli G. Tiwari V. (2018). Strategies for combating bacterial biofilms: a focus on anti-biofilm agents and their mechanisms of action. Virulence 9 (1), 522–554. 10.1080/21505594.2017.1313372 28362216 PMC5955472

[B23] Seil J. T. Webster T. J. (2012). Antimicrobial applications of nanotechnology: methods and literature. Int. J. Nanomedicine 7, 2767–2781. 10.2147/IJN.S24805 22745541 PMC3383293

[B24] Theuretzbacher U. Gottwalt S. Beyer P. Butler M. Czaplewski L. Lienhardt C. (2019). Analysis of the clinical antibacterial and antituberculosis pipeline. Lancet Infect. Dis. 19 (2), e40–e50. 10.1016/S1473-3099(18)30513-9 30337260

[B25] Tilley S. J. Orlova E. V. Gilbert R. J. Andrew P. W. Saibil H. R. (2005). Structural basis of pore formation by the bacterial toxin pneumolysin. Cell. 121 (2), 247–256. 10.1016/j.cell.2005.02.033 15851031

[B26] Vila J. Moreno-Morales J. Ballesté-Delpierre C. (2020). Current landscape in the discovery of novel antibacterial agents. Clin. Microbiol. Infect. 26 (5), 596–603. 10.1016/j.cmi.2019.09.015 31574341

[B27] Weiser J. N. Ferreira D. M. Paton J. C. (2018). Streptococcus pneumoniae: transmission, colonization and invasion. Nat. Rev. Microbiol. 16 (6), 355–367. 10.1038/s41579-018-0001-8 29599457 PMC5949087

[B28] Xu M. Y. Kim Y. S. (2014). Antitumor activity of glycyrol via induction of cell cycle arrest, apoptosis and defective autophagy. Food Chem. Toxicol. 74, 311–319. 10.1016/j.fct.2014.10.023 25445757

[B29] Zafar M. A. Wang Y. Hamaguchi S. Weiser J. N. (2017). Host-to-Host transmission of Streptococcus pneumoniae is driven by its inflammatory toxin, pneumolysin. Pneumolysin. Cell Host Microbe 21 (1), 73–83. 10.1016/j.chom.2016.12.005 28081446 PMC5267320

[B30] Zhang L. Zeng X. Guo D. Zou Y. Gan H. Huang X. (2022). Early use of probiotics might prevent antibiotic-associated diarrhea in elderly (>65 years): a systematic review and meta-analysis. BMC Geriatr. 22 (1), 562. 10.1186/s12877-022-03257-3 35794520 PMC9260993

